# Entre normas e práticas: implementação e repercussões regionais de mudanças nas políticas de atenção primária à saúde no Brasil

**DOI:** 10.1590/0102-311XPT206924

**Published:** 2025-09-01

**Authors:** Luciana Dias de Lima, Henrique Sant’Anna Dias, Fernanda de Freitas Mendonça, André Schimidt da Silva, Brígida Gimenez Carvalho, Caroline Pagani Martins, Adelyne Maria Mendes Pereira

**Affiliations:** 1 Escola Nacional de Saúde Pública Sergio Arouca, Fundação Oswaldo Cruz, Rio de Janeiro, Brasil.; 2 Universidade Estadual de Londrina, Londrina, Brasil.

**Keywords:** Atenção Primária à Saúde, Política de Saúde, Estratégias de Saúde Nacionais, Saúde da Família, Sistema Único de Saúde, Primary Health Care, Health Policy, National Health Strategies, Family Health, Unified Health System, Atención Primaria de Salud, Política de Salud, Estrategias de Salud Nacionales, Salud de la Familia, Sistema Único de Salud

## Abstract

O artigo analisa a implementação de mudanças nas políticas nacionais da atenção primária à saúde (APS) no Brasil, e suas repercussões na região de saúde Metropolitana I do Rio de Janeiro e na Macrorregional Norte do Paraná, entre 2017 e 2022. De natureza multicêntrica, o estudo adota abordagem comparativa e métodos mistos para identificar semelhanças e diferenças entre os casos em três eixos analíticos: perfil, posicionamento institucional e concepções sobre a APS; decisões organizacionais do processo de trabalho; e cobertura e composição das equipes. Foram realizadas 44 entrevistas com gestores em 14 municípios selecionados e analisados dados secundários. A formação superior na saúde, a inserção na Saúde Coletiva e o tempo curto no cargo, decorrente das eleições municipais, foram características comuns dos entrevistados. Observou-se convergência nas concepções sobre a APS e a Estratégia Saúde da Família, além de tensões e posicionamentos favoráveis ao Programa Previne Brasil, que incentivou mudanças organizacionais no cadastramento da população e no cumprimento de metas. Identificou-se diversidade de estratégias de monitoramento, avaliação e manutenção de programas e ações, redução de agentes comunitários de saúde por equipe e aumento de equipes por unidade básica. Apesar da predominância das equipes de saúde da família, houve diferenças na proporção de população cadastrada e no número de equipes do Núcleo de Apoio à Saúde da Família. A análise sugere que a implementação das diretrizes da APS e suas repercussões são influenciadas por fatores complexos e interdependentes, como capacidades municipais, relações intergovernamentais e atuação estadual no contexto regional.

## Abstract (en)

This article analyzes the implementation of changes in national primary health care (PHC) policies in Brazil, and their repercussions in the Metropolitan Health Region I of Rio de Janeiro and in the Northern MacroRregion of Paraná between 2017 and 2022. This multicenter study adopts a comparative approach and mixed methods to identify similarities and differences between cases in three analytical axes: profile, institutional positioning, and conceptions about PHC; organizational decisions related to the work process; coverage and composition of teams. A total of 44 interviews were conducted with managers in 14 selected municipalities and secondary data were analyzed. Higher degrees in health, introduction to Public Health and short time in office, due to municipal elections, were common characteristics of the interviewees. There was convergence in the conceptions of PHC and the Family Health Strategy, as well as tensions and favorable positions for the Prevent Brazil Program, which encouraged organizational changes in the registration of the population and in the fulfillment of goals. A diverse set of strategies for monitoring, evaluation and maintenance of programs and actions was identified, as well as a reduction in the number of community health agents per team and an increase in the number of teams per basic health unit. Despite the predominance of family health teams, there were differences between the proportion of the registered population and the number of teams in the Family Health Support Center. The analysis suggests that the implementation of PHC guidelines and their repercussions are influenced by complex and interdependent factors, such as municipal capacities, intergovernmental relations, and state action within the regional context.

## Abstract (es)

El artículo analiza la implementación de cambios en las políticas nacionales de atención primaria de salud (APS) en Brasil y sus repercusiones en la región sanitaria Metropolitana I de Río de Janeiro y en la Macrorregión Norte de Paraná, entre 2017 y 2022. Este estudio multicéntrico adopta un enfoque comparativo y métodos mixtos para identificar similitudes y diferencias entre los casos en tres ejes analíticos: perfil, posicionamiento institucional y concepciones sobre la APS; decisiones organizativas del proceso de trabajo; cobertura y composición de los equipos. Se realizaron 44 entrevistas con gestores de 14 municipios seleccionados y se analizaron datos secundarios. La formación superior en salud, la inserción en Salud Colectiva y el breve tiempo en el cargo, debido a las elecciones municipales, fueron características comunes de los entrevistados. Se observó una convergencia en las concepciones sobre la APS y la Estrategia de Salud de la Familia, además de tensiones y posiciones favorables al Programa Previne Brasil, que impulsó cambios organizativos en el registro de la población y en el cumplimiento de metas. Se identificó diversidad en las estrategias de monitoreo, evaluación y mantenimiento de programas y acciones, reducción de agentes comunitarios de salud por equipo y aumento de equipos por unidad básica. A pesar del predominio de los equipos de salud de la familia, hubo diferencias en la proporción de población registrada y en el número de equipos del Núcleo de Apoyo a la Salud de la Familia. El análisis sugiere que la implementación de las directrices de la APS y sus repercusiones están influenciadas por factores complejos e interdependientes, como las capacidades municipales, las relaciones intergubernamentales y la actuación estatal en el contexto regional.

## Introdução

A atenção primária à saúde (APS) é central na configuração de sistemas públicos e universais, que possuem variações quanto à sua abrangência, cobertura assistencial e integração do cuidado ^1^. No Brasil, desde a segunda metade dos anos 1990, a Estratégia Saúde da Família (ESF) consolidou-se como referência para a organização da APS no Sistema Único de Saúde (SUS) ^2^.

Entre os aspectos desse modelo, destaca-se as equipes multiprofissionais no cuidado individual, familiar e coletivo/comunitário, de modo a atender às necessidades de saúde diversas ^3^. Estudos indicam que a implantação e a expansão da ESF contribuíram para melhoria da oferta e do acesso aos cuidados primários ^4^, e favoreceram a redução da mortalidade infantil ^5^ e de internações evitáveis ^6^.

A difusão nacional da ESF pode ser explicada pela prioridade que adquiriu na agenda do Ministério da Saúde nos anos 2000. Nesse cenário, ressalta-se a criação de programas e políticas nacionais, envolvendo modalidades de repasses de recursos federais que induziram sua implantação pelos municípios, com efeitos redistributivos para regiões mais carentes ^7^.

Entretanto, a configuração da APS e a implantação da ESF não seguiram um padrão homogêneo, dadas as desigualdades e diversidades do território brasileiro. Municípios de grande porte populacional enfrentam desafios para estruturar a APS ^8^ ^,^ ^9^, especialmente nas metrópoles ^10^. Já os municípios de pequeno porte, embora apresentem melhor cobertura na atenção primária, lidam com barreiras de acesso relacionadas a condições geográficas e organizacionais ^11^ ^,^ ^12^.

A partir de 2017, mudanças na Política Nacional de Atenção Básica (PNAB 2017) flexibilizaram parâmetros de composição e cobertura das equipes da ESF, acarretando retrocessos e riscos para sua organização no SUS ^13^. Em 2019, o Programa Previne Brasil introduziu novas regras de financiamento da APS, orientando os repasses federais com base no cadastramento da população, implantação de programas específicos e cumprimento de indicadores de desempenho ^14^ ^,^ ^15^.

Esses mecanismos substituíram a lógica alocativa baseada em valores *per capita* conforme classificação socioeconômica dos municípios. O incentivo para as equipes dos Núcleos de Apoio à Saúde da Família (NASF) foi extinto, adotando-se critérios de remuneração que favoreceram a redução do número de agentes comunitários de saúde (ACS) e promoveram a formação de equipes multiprofissionais distintas do modelo ESF: as equipes de atenção primária (EqAP) ^16^. As perdas de recursos e a instabilidade nos repasses federais, somadas aos desafios de cadastramento e monitoramento de indicadores, repercutiram no financiamento e na gestão municipal da APS ^17^ ^,^ ^18^.

O objetivo do estudo foi analisar a implementação das mudanças contidas na PNAB 2017 e no Previne Brasil 2019, e suas repercussões sobre o modelo de APS em municípios e regiões selecionadas. Partimos do pressuposto de que a implementação de políticas nacionais em nível locorregional é afetada pelas particularidades contextuais e dos atores envolvidos, com variações de operacionalização e desfechos observados entre unidades federativas e regiões de saúde no Brasil.

## Método

Trata-se de estudo multicêntrico, que adota abordagem comparativa e aprofundada de poucos casos, visando identificar semelhanças e diferenças entre eles. A pesquisa abrangeu duas regiões contrastantes, que abrigam os atores e instituições responsáveis pela implementação das políticas em foco: a região de saúde Metropolitana I do Rio de Janeiro, com cerca de 10 milhões de habitantes em 12 municípios de médio e grande porte populacional; e a Macrorregional Norte do Paraná, composta por cinco regiões de saúde, com aproximadamente 2 milhões de habitantes distribuídos em 97 municípios, predominantemente de pequeno porte populacional.

Foram selecionados 14 municípios, com perfis diferenciados quanto à dependência de repasses federais para a APS, sendo cinco da região Metropolitana I do Rio de Janeiro e nove da Macrorregional Norte do Paraná. A seleção envolveu ranqueamento dos municípios em quartis em cada região, com base na média do indicador “proporção de transferências federais da APS nas despesas municipais com ações e serviços públicos de saúde (ASPS)” no período de 2015-2020. Esse indicador foi calculado a partir de dados do Fundo Nacional de Saúde (https://portalfns.saude.gov.br/) e do Sistema de Informações de Orçamentos Públicos em Saúde (https://www.gov.br/saude/pt-br/acesso-a-informacao/siops). Foram incluídos os núcleos urbanos mais relevantes de cada região: Rio de Janeiro, capital do estado, e Londrina (Paraná).

A Tabela 1 apresenta a caracterização dos municípios selecionados por regiões de saúde, segundo indicadores sociodemográficos e de dependência de repasses federais do SUS para a APS, como expressão da diversidade de situações municipais encontradas.

**Tabela 1:** Caracterização dos municípios por região de saúde segundo indicadores selecionados.

Região de saúde/Município	Porte populacional	IDH-M	**PIB *per capita* (R$)**	Esgotamento sanitário adequado (%)	Transferências federais de APS nas despesas municipais com ASPS (%) *
Metropolitana I do Rio de Janeiro					
Duque de Caxias	Grande	0,799	49.122,8	94,4	7,0
Mesquita	Grande	0,711	51.041,9	85,3	5,9
Nova Iguaçu	Grande	0,713	20.904,6	83,1	12,8
Rio de Janeiro	Grande	0,737	14.331,5	93,3	8,0
Seropédica	Médio	0,713	55.720,8	64,1	19,4
Macrorregional Norte do Paraná					
Apucarana	Grande	0,748	24.493,1	41,6	14,6
Arapuã	Pequeno	0,676	42.450,9	9,2	20,4
Cândido de Abreu	Pequeno	0,629	26.109,6	18,9	20,5
Conselheiro Mairinck	Pequeno	0,707	30.422,6	69,1	13,4
Cruzmaltina	Pequeno	0,666	54.941,8	39,2	8,4
Londrina	Grande	0,778	37.715,1	85,2	8,0
Pinhalão	Pequeno	0,697	29.740,5	7,7	23,8
Porecatu	Pequeno	0,738	51.359,2	95,3	8,0
Santo Antônio do Paraíso	Pequeno	0,716	44.150,0	2,5	18,5

APS: atenção primária à saúde; ASPS: ações e serviços públicos de saúde; IDH-M: Índice de Desenvolvimento Humano Municipal; PIB: produto interno bruto.

Fontes: porte populacional ^34^; IDH-M ^35^; PIB *per capita* ^36^; esgotamento sanitário ^37^.

* Proporção de transferências federais da APS nas despesas municipais com ASPS, média 2015-2020: Sistema de Informações sobre Orçamentos Públicos em Saúde e Fundação Nacional de Saúde.

O estudo se ancora em marco analítico que considera as interdependências entre os níveis de governo e a circulação de ideias, modelos e práticas entre os entes, no contexto federativo brasileiro ^19^. Também incorporou perspectivas sobre o papel dos atores locais, cujas interpretações, capacidades institucionais e decisões influenciam a forma como as diretrizes nacionais são implementadas ^20^.

Com base nesses referenciais, foram adotados métodos mistos para coleta, sistematização e análise das informações em três eixos: (1) perfil, posicionamento institucional e concepções sobre a APS; (2) decisões relacionadas à organização do processo de trabalho na APS; e (3) cobertura e composição das equipes de APS. O período, de 2017 a 2022, corresponde à vigência das diretrizes da PNAB 2017 e do Previne Brasil 2019.

Os dois primeiros eixos envolveram 44 entrevistas com atores-chave da gestão da APS, realizadas entre junho de 2022 e fevereiro de 2023. Os entrevistados dividiram-se em dois perfis: (1) gestores e tomadores de decisão municipais - secretários, subsecretários e coordenadores de APS (33 entrevistados: oito no Rio de Janeiro e 25 no Paraná); e (2) técnicos e dirigentes de organizações de gestão estadual, regional e de representação de gestores municipais - Secretaria de Estado de Saúde (SES), Conselho de Secretários Municipais de Saúde (COSEMS) e consórcios de saúde regionais (11 entrevistados: sete no Rio de Janeiro e quatro no Paraná).

O primeiro eixo focou na formação e trajetória dos atores, incluindo especificação e tempo no cargo, concepções sobre a APS, especialmente a ESF, e posicionamentos sobre as mudanças trazidas pela PNAB 2017 e o Previne Brasil 2019. O segundo eixo enfatizou as decisões sobre adesão e manutenção de programas e estratégias nacionais, as mudanças organizacionais e as estratégias de monitoramento e avaliação.

As entrevistas seguiram roteiro para cada perfil, foram gravadas com autorização e transcritas. O conteúdo das entrevistas passou por pré-análise, exploração e categorização temática, sendo interpretado conforme os pressupostos e o referencial analítico do estudo.

O terceiro eixo envolveu análise descritiva de dados do Sistema de Informação em Saúde para a Atenção Básica (SISAB - https://sisab.saude.gov.br/), calculando-se a proporção da população cadastrada de 2018 (primeiro ano com dados disponíveis) a 2022, por município, média e variação do período. Utilizou-se estimativas populacionais do Instituto Brasileiro de Geografia e Estatística (IBGE - https://www.ibge.gov.br/) para o Tribunal de Contas da União (TCU), exceto para 2022, para o qual se adotou a estimativa de 2021. Esse procedimento foi necessário para evitar distorções nos indicadores da série histórica, pela redução populacional observada no *Censo Demográfico* 2022 e da ausência de dados corrigidos das estimativas para os anos anteriores.

Nesse eixo, também coletou-se dados do Cadastro Nacional de Estabelecimentos de Saúde (CNES - https://cnes.datasus.gov.br/), referentes ao número de equipes na APS - incluindo NASF, ACS, EqAP, equipes de saúde da família (EqSF), equipes de saúde bucal (EqSB) - e unidades básicas de saúde (UBS). Foram calculadas as médias e a variação de 2017 a 2022. Os dados foram coletados em dezembro/2023.

O Quadro 1 sistematiza o referencial analítico e as fontes de informação da pesquisa.

O estudo foi aprovado pelo Comitê de Ética em Pesquisa da Escola Nacional de Saúde Pública Sergio Arouca, Fundação Oswaldo Cruz (CAAE: 30675420.6.1001.5240).

Os achados dos eixos 1 e 2 são apresentados separadamente para cada caso. Já o eixo 3 integra ambos os contextos investigados. A discussão final explora comparativamente os resultados, evidenciando semelhanças, contrastes e particularidades entre as regiões.

**Quadro 1:** Componentes, indicadores e fontes de informação segundo eixos de análise.

EIXOS DE ANÁLISE	COMPONENTES/INDICADORES	FONTES DE INFORMAÇÃO
Perfil, posicionamento institucional e concepções dos gestores da APS	Formação e trajetória na saúde Função e tempo no cargo Concepções sobre: a APS, a ESF, a PNAB 2017 e o Programa Previne Brasil 2019	Entrevistas com gestores
Decisões relacionadas à organização do processo de trabalho na APS	Adesão/manutenção de programas e ações estratégicas nacionais para a APS Alterações de processos organizacionais na APS a partir da PNAB 2017 e Previne Brasil 2019 Estratégias e instrumentos para o monitoramento e avaliação da APS	Entrevistas com gestores
Cobertura e composição da APS	% de população cadastrada Número de equipes NASF credenciadas Número de ACS Número de EqAP Número de EqSF Número de EqSB Número de UBS * Média de ACS por EqAP e EqSF Média de equipes por UBS	Dados secundários obtidos do SISAB, SCNES e Instituto Brasileiro de Geografia e Estatística

ACS: agentes comunitários de saúde; APS: atenção primária à saúde; EqAP: equipe de atenção primária; EqSB: equipe de saúde bucal; EqSF: equipe de saúde da família; ESF: Estratégia Saúde da Família; NASF: Núcleos de Apoio à Saúde da Família; PNAB: Política Nacional de Atenção Básica; SCNES: Sistema do Cadastro Nacional de Estabelecimentos de Saúde; SISAB: Sistema de Informação em Saúde para a Atenção Básica; UBS: unidade básica de saúde.

* Contempla os seguintes estabelecimentos: posto de saúde, centro de saúde/unidade básica, unidade mista, unidade de saúde da família, centro de apoio à saúde da família.

## Resultados

### Perfis, concepções e decisões organizacionais dos gestores na região de saúde Metropolitana I do Rio de Janeiro

Na região de saúde Metropolitana I do Rio de Janeiro, a maioria dos gestores municipais possuía formação superior em saúde. Apenas um dirigente relatou formação técnica em gestão de sistemas e serviços de saúde. Entre os oito entrevistados, quatro eram da área de odontologia (um com segunda graduação em medicina), seguidos por medicina (2) e enfermagem (1). Pós-graduações em Saúde Coletiva e Saúde da Família foram comuns entre os coordenadores de APS, alguns com pós-graduação inicial em áreas clínicas, como medicina e odontologia.

As trajetórias na saúde variaram, com atuação prévia em cargos de gestão. Essas experiências incluíram coordenações de programas vinculados à APS, como Saúde na Escola, Saúde Bucal, Academia da Saúde e NASF, e atuação em serviços municipais de atenção primária e hospitalar.

Os entrevistados das representações estaduais e regionais (SES-RJ, COSEMS RJ e Consórcio Intermunicipal de Saúde da Baixada Fluminense - CISBAF) possuíam perfil de nível superior na área da saúde com especialização, residência ou mestrado em áreas relacionadas à APS, Saúde Coletiva, Gestão e Planejamento em Saúde; e inserção diversa no SUS, em atividades assistenciais e de gestão no âmbito nacional, estadual, regional e local. Entre eles, havia relevante variação do tempo de exercício das funções desempenhadas em suas trajetórias prévias, algumas superiores a 20 anos.

O tempo na função gestora exercida no momento da entrevista era predominantemente curto (menos de um ano a pouco mais de dois anos) especialmente entre os gestores municipais, em alinhamento com o ingresso a partir das eleições municipais. Na SES-RJ e COSEMS RJ, o tempo nas funções era igualmente curto, ao contrário do CISBAF, em que a gestão foi mais estável.

A visão sobre APS destacava sua importância como modelo robusto e abrangente, com diferentes perspectivas sobre coordenação do cuidado, resolutividade e territorialização. A ESF foi associada ao arranjo mais eficaz, especialmente devido ao papel dos ACS e das equipes multiprofissionais. Críticas sobre o “modelo tradicional” de APS foram também relatadas.

As concepções se diferenciaram pelo grau de familiaridade com as diretrizes e os conteúdos dos atos normativos federais sobre a APS; e a presença de um olhar mais acadêmico de atores que, embora na gestão, trilharam caminho formativo mais duradouro no ambiente universitário e da pesquisa em Saúde Coletiva. Observou-se ponderações dos limites do modelo da ESF, em razão de problemas na formação, no financiamento e na baixa priorização da APS pela gestão.

Predominou-se uma visão municipal favorável ao Previne Brasil, com foco no controle do registro da produção dos serviços visando melhores resultados em indicadores de desempenho, embora tenham sido relatados receios quanto às capacidades institucionais frente às novas demandas. As preocupações com perdas dos recursos federais associaram-se aos esforços da SES-RJ e do COSEMS RJ em instrumentalizar os municípios nos cálculos de valores e nos resultados de indicadores preconizados pelo programa. Foram residuais as menções sobre mudanças introduzidas pela PNAB 2017.

A proposta do Previne Brasil 2019 mobilizou o COSEMS RJ ainda antes de aprovada na Comissão Intergestores Tripartite (CIT), com preocupações sobre o eventual impacto financeiro para os municípios, bem como a distorção do modelo de atenção. Entre os entrevistados, alguns apoiaram e outros criticaram iniciativas conjuntas do COSEMS RJ e da SES-RJ por identificarem uma oposição política ao Governo Federal, defendendo que, ao invés disso, os esforços buscassem ajustar pontos críticos do programa.

Os municípios desenvolveram ações de preservação das equipes NASF credenciadas em resposta à perda do incentivo federal via incentivo específico estabelecido pelo Programa Estadual de Financiamento da APS, da SES-RJ. Ainda assim, foi relatada redução do número de profissionais, bem como deslocamento de trabalhadores para outros serviços de saúde.

A principal mudança no processo de trabalho associou-se ao cadastramento populacional no e-SUS, para ampliar recursos do componente capitação ponderada do modelo de financiamento do Previne Brasil 2019. Outras mudanças relacionaram-se à demanda por qualificação do registro da produção e à preocupação com a informação produzida nos serviços, dada a necessidade de calcular indicadores para a captação de recursos federais, ponto-chave do componente de avaliação de desempenho.

Os municípios implementaram diferentes iniciativas para profissionais se qualificarem nos registros de produção. Em alguns casos, foram designados técnicos para atuar nas unidades e digitadores centralizados nas secretarias municipais para suprir lacunas tecnológicas.

Houve opiniões divergentes sobre prontuários eletrônicos privados que, embora fornecessem relatórios gerenciais adequados à gestão municipal, apresentaram falhas na transferência completa dos dados ao Governo Federal, prejudicando o cumprimento das metas do Previne Brasil. A maioria dos municípios usou exclusivamente o e-SUS para registro da produção.

Para os gestores municipais, essas mudanças fortaleceram os processos de monitoramento e avaliação de desempenho na APS, favorecidos pelo apoio técnico oferecido pela SES-RJ e COSEMS RJ.

### Perfis, concepções e decisões organizacionais dos gestores na Macrorregional Norte do Paraná

Na Macrorregional Norte do Paraná, dos nove secretários de saúde, sete possuíam ensino superior, sendo cinco na área da saúde (odontologia [1], farmácia [1] e enfermagem [3]) e dois em pedagogia; os outros dois tinham ensino médio e fundamental. Cinco secretários mencionaram pós-graduação, principalmente em Saúde Coletiva, incluindo mestrado e doutorado (em curso). A maioria assumiu o cargo após as eleições de 2020, exceto um, cujo tempo na função era de pelo menos 20 anos. Mesmo aqueles com pouco tempo no cargo possuíam alguma vivência prévia de gestão em áreas e/ou localidades distintas.

Entre os 16 subsecretários e coordenadores de APS entrevistados, predominou a graduação em enfermagem (11), com dois formados em medicina, um em pedagogia e dois sem nível superior. A formação *lato sensu* em Saúde Coletiva, Saúde da Família e Gestão Pública foi expressiva, enquanto a formação stricto sensu em Saúde Coletiva foi relatada por apenas duas entrevistadas. O tempo de experiência na saúde variou de um ano e meio a mais de 30 anos, com os períodos mais longos associados a servidores públicos (10). Poucos relataram experiência prévia em gestão.

Os quatro entrevistados das representações estaduais e regionais tinham formação na área da saúde, com predomínio da enfermagem (3) e um psicólogo. Todos possuíam pós-graduação em gestão pública e áreas correlatas, e ampla experiência em cargos de gestão municipal e/ou estadual.

As concepções sobre APS pouco variaram. Todos os entrevistados enfatizaram a importância desse nível assistencial para promoção da saúde e prevenção de agravos, compreendendo-o como o serviço mais próximo do usuário e resolutivo. Entre representantes estaduais e regionais, prevaleceu a visão de concretização da integralidade somente a partir da APS.

O modelo da ESF foi considerado essencial para organizar a APS nos territórios, compatibilizando-a com práticas de territorialização, identificação de grupos de risco, visitas domiciliares e presença dos ACS e das equipes multiprofissionais. No entanto, em três municípios relataram-se estratégias de atendimento médico por demanda espontânea, secundarizando ações coletivas, multiprofissionais e de base territorial.

Observou-se desconhecimento da maioria dos gestores municipais sobre as mudanças trazidas pela PNAB 2017. Alguns entrevistados das representações estaduais e regionais argumentaram que a PNAB 2017 deu maior flexibilidade aos gestores municipais ao introduzir o financiamento para as EqAP e ampliar o escopo de atuação das equipes multiprofissionais.

Em relação ao Previne Brasil 2019, prevaleceu um posicionamento favorável, mesmo reconhecendo os riscos de redução de recursos. O pagamento por desempenho foi defendido por favorecer a qualificação dos registros. Considerou-se relevante a capitação ponderada para incentivar o cadastramento da população. Entre as representações estaduais e regionais, houve tom mais crítico, considerando que o Previne Brasil 2019 dominou a agenda política dos municípios, enfraquecendo a discussão mais ampla sobre a APS.

O processo de trabalho das equipes NASF foi descontinuado em dois municípios. Um terceiro relatou nunca ter credenciado. A manutenção dos NASF em quatro municípios foi possível com recursos próprios. O apoio matricial vinculado ao processo de trabalho foi citado apenas nos dois maiores municípios, que contavam com residência em saúde da família.

A implantação do Previne Brasil 2019 gerou mudanças significativas para evitar a perda de recursos federais, adotadas independentemente do porte populacional ou das características das equipes. Tais mudanças incluíram a implementação do prontuário eletrônico, treinamento das equipes, (re)cadastramento segundo a capitação ponderada, reorganização dos fluxos de atendimento e monitoramento dos indicadores.

As estratégias de (re)cadastramento da população incluíram mutirões nos finais de semana, busca ativa na zona rural sem ACS residentes e uso de *tablets* para qualificar o cadastro.

Em relação ao prontuário eletrônico, a adesão ao e-SUS ocorreu apenas nos municípios com equipes de apoio mais estruturadas ou com profissionais na APS experientes no sistema. Por sua vez, aqueles que adquiriram sistemas privados relataram benefícios, como capacitação e apoio na correção de inconsistências.

Nos municípios com sistemas privados, o monitoramento dos indicadores era mensal, enquanto nos que usavam o e-SUS era quadrimestral. Apenas dois monitoraram indicadores além daqueles do Previne Brasil 2019, incorporando outros sistemas nacionais de informação. Falta de apoio técnico, dificuldades com o prontuário eletrônico e limitações de acesso à internet representaram desafios adicionais para a efetivação dessa atividade.

### População cadastrada, evolução das equipes e das UBS nas regiões

Entre 2018 e 2022, a média da proporção da população cadastrada no SISAB foi de 43,5% na região Metropolitana I do Rio de Janeiro e de 70% na Macrorregional Norte do Paraná. Em ambos os casos, houve aumento de cadastros no período, embora o Estado do Paraná e a Macrorregião Norte tenham apresentado médias mais altas (72,5% e 70%, respectivamente) em comparação ao Estado do Rio de Janeiro (44,6%) e à Metropolitana I (43,5%).

Observa-se, no entanto, um crescimento mais expressivo para o Rio de Janeiro, onde a cobertura na Metropolitana I aumentou de 19%, em 2018, para 65,6%, em 2022, um crescimento de 245,8%. Na Macrorregional Norte do Paraná, a cobertura passou de 50,7%, em 2018, para mais de 90%, em 2022, atingindo 78,5% no período (Tabela 2).

**Tabela 2:** Proporção de população cadastrada de municípios e regiões selecionadas dos estados do Rio de Janeiro e do Paraná, Brasil, 2018 a 2022.

Localidade	2018	2019	2020	2021	2022	X̅ *	Variação * (%)
Rio de Janeiro	22,4	28,8	46,8	58,8	66,0	44,6	195,3
Região Metropolitana I	19,0	26,2	48,6	58,3	65,6	43,5	245,8
Duque de Caxias	4,5	4,9	6,3	16,5	21,7	10,8	387,7
Mesquita	20,1	30,7	51,0	73,8	88,9	52,9	341,2
Nova Iguaçu	10,5	15,5	22,1	27,9	34,8	22,2	232,1
Rio de Janeiro	22,5	31,3	63,0	72,7	79,6	53,8	253,9
Seropédica	23,3	27,6	39,1	56,0	70,2	43,2	201,6
Paraná	50,1	60,7	74,0	86,0	91,6	72,5	82,8
Macrorregião Norte	50,7	55,7	68,4	84,5	90,5	70,0	78,5
Apucarana	66,1	71,1	76,6	87,1	95,1	79,2	43,8
Arapuã	67,9	76,2	91,4	100,0	100,0	87,1	47,3
Cândido de Abreu	71,5	82,0	89,1	95,8	100,0	87,7	39,8
Conselheiro Mairinck	33,8	32,7	57,5	98,9	100,0	64,6	195,8
Cruzmaltina	100,0	100,0	100,0	100,0	100,0	100,0	0,0
Londrina	17,4	19,3	38,9	59,7	65,8	40,2	277,7
Pinhalão	95,3	99,0	95,0	100,0	100,0	97,9	4,9
Porecatu	70,4	75,8	82,0	91,7	95,0	83,0	34,8
Santo Antônio do Paraíso	70,3	84,4	85,6	100,0	100,0	88,1	42,3

X̅: média.

Fonte: Sistema de Informação em Saúde para a Atenção Básica (SISAB, https://sisab.saude.gov.br/) e Instituto Brasileiro de Geografia e Estatística - estimativas de população.

* Período de 2018-2022.

As variações percentuais de população cadastrada nos municípios foram superiores a 200% na Metropolitana I do Rio de Janeiro, variando de 201,6% a 387,7%, já no Paraná, entre 30% e 40%. No Rio de Janeiro, os percentuais iniciais eram menores (máximo de 23,3%), enquanto no Paraná a maioria dos municípios tinha percentuais mais altos em 2018. Em 2022, seis dos nove municípios do Paraná alcançaram 100% de população cadastrada, proporção não observada no Rio de Janeiro, onde o máximo foi 88,9% (Tabela 2).

Quanto à capacidade instalada da APS, o aumento no total de EqSF, entre 2017 e 2022, foi maior do que o incremento no número de ACS, reduzindo a média de ACS por EqSF, tanto no Rio de Janeiro (queda de 3,8% na Metropolitana I e de 8,2% no estado), quanto no Paraná (queda de 6,2% na Macrorregião Norte e de 11,5% no estado). Três municípios da Metropolitana I e dois da Macrorregião Norte apresentaram uma redução superior a 15% na média de ACS por EqSF. Além disso, o número de UBS não acompanhou a expansão de EqSF, aumentando as médias de e-SF por UBS em 12% na Metropolitana I e em 4,1% na Macrorregional Norte (Tabela 3).

**Tabela 3:** Média (x̅) e variação proporcional (%) da capacidade instalada na atenção primária à saúde em regiões selecionadas. Brasil, 2017 a 2022.

Localidade	EqSF		ACS		ACS/EqSF		UBS		EqSF/UBS		EqAP		EqSB		NASF	
	x̅	%	x̅	%	x̅	%	x̅	%	x̅	%	x̅	%	x̅	%	x̅	%
Rio de Janeiro	3.048	18,4	17151	8,7	5,6	-8,2	2050	5,0	1,5	12,8	124	871,4	1377	29,4	219	31,7
Metropolitana I	1.647	17,6	9343	13,1	5,7	-3,8	569	5,0	2,9	12,0	57	288,0	546	15,4	112	23,8
Duque de Caxias	73	15,7	374	-15,7	5,2	-27,1	47	8,7	1,5	6,5	4	-	37	45,5	3	0,0
Mesquita	36	173,7	164	47,3	4,9	-46,2	18	-41,7	2,3	369,2	0	-	13	257,1	3	-
Nova Iguaçu	116	34,4	642	-13,5	5,6	-35,7	59	1,7	2,0	32,2	8	-	29	11,5	14	7,7
Rio de Janeiro	1.154	11,6	5637	14,4	4,9	2,5	262	0,8	4,4	10,8	42	128,0	403	11,1	81	23,1
Seropédica	22	0,0	151	3,3	6,8	3,3	20	5,3	1,1	-5,0	0	-	16	0,0	1	0,0
Paraná	2.548	14,0	13073	0,9	5,1	-11,5	2703	1,4	0,9	12,4	227	635,6	1573	49,5	306	0,0
Macrorregião Norte	535	5,7	2619	-0,8	4,9	-6,2	524	1,5	1,0	4,1	11	-	294	13,8	74	-1,4
Apucarana	43	0,3	190	2,1	4,5	-0,2	32	3,1	1,3	-0,8	-	-	25	0,0	4	0,0
Arapuã	2	0,0	13	10,0	6,5	10,0	4	-60,0	0,6	150,0	-	-	2	0,0	0	-
Cândido de Abreu	6	0,0	42	-6,8	7,0	-6,8	13	0,0	0,5	0,0	-	-	4	0,0	0	-
Conselheiro Mairinck	1	0,0	8	0,0	7,5	0,0	1	-50,0	0,9	100,0	-	-	1	0,0	0	-
Cruzmaltina	1	0,0	8	-22,2	6,3	-22,2	3	0,0	0,4	0,0	1	-	0	-	1	0,0
Londrina	109	7,6	282	-10,5	2,6	-16,8	57	3,6	1,9	3,9	11	-	31	-9,1	10	0,0
Pinhalão	3	0,0	15	-6,3	5,1	-6,2	5	-33,3	0,6	50,0	-	-	2	0,0	0	-
Porecatu	4	0,0	20	-9,5	5,3	-9,5	4	0,0	1,0	0,0	-	-	3	0,0	1	0,0
Santo Antônio do Paraíso	1	0,0	9	11,1	9,2	11,1	2	100,0	0,7	-50,0	-	-	0	-100,0	0	-

ACS/EqSF: média do número de agentes comunitários de saúde por equipe de saúde da família; EqAP: equipes de atenção básica; EqSB: equipes de saúde bucal; EqSF/UBS: média do número de equipes de saúde da família por unidade básica de saúde; NASF: Núcleos de Apoio à Saúde da Família.

Fonte: Ministério da Saúde - Cadastro Nacional dos Estabelecimentos de Saúde (CNES).

A coexistência de equipes diferentes da EqSF foi residual nas regiões, ocorrendo em três municípios da Metropolitana I e em dois da Macrorregional Norte. Com o pequeno número de EqAP, as variações percentuais, quando presentes, foram expressivas, com 288% na Metropolitana I, 871,4% no Estado do Rio de Janeiro e 635,6% no Paraná (sem variação na Macrorregião Norte). Em âmbito municipal, observou-se essa variação nas EqAP no Município do Rio de Janeiro, que registrou aumento de 128% dessas equipes entre 2017 e 2022 (Tabela 3).

Ambas as regiões apresentaram padrão semelhante na evolução das EqSB, com aumento de 13,8% na Macrorregional Norte do Paraná e 15,4% na Metropolitana I do Rio de Janeiro, valores abaixo dos respectivos estados (49,5% no Paraná e 29,4% no Rio de Janeiro). A maior diferença entre as regiões ocorreu em relação ao NASF: cinco dos nove municípios da Macrorregião Norte não tinham equipes NASF, com uma leve redução percentual entre 2017 e 2022, enquanto na Metropolitana I todos os municípios possuíam ao menos uma equipe NASF, com expansão no período (Tabela 3).

O Quadro 2 sintetiza as características da implementação e das repercussões das mudanças na configuração e no financiamento da APS nas regiões, considerando os eixos de análise deste estudo.

**Quadro 2:** Implementação e repercussões das mudanças contidas na Política Nacional de Atenção Básica (PNAB) de 2017 e nas orientações do Programa Previne Brasil 2019 em regiões selecionadas dos estados do Rio de Janeiro e do Paraná, Brasil.

EIXOS DE ANÁLISE	REGIÃO METROPOLITANA I DO RIO DE JANEIRO	MACRORREGIÃO NORTE DO PARANÁ
Perfil, posicionamento institucional e concepções dos gestores da APS	Maioria dos gestores municipais com formação em áreas da saúde, com trajetórias diversificadas e experiências em gestão prévia, especialmente na Baixada Fluminense Representantes estaduais e regionais com qualificações avançadas e vasta experiência em saúde pública e gestão, com alguns tendo carreiras superiores a 20 anos O período na função gestora foi curto para a maioria dos gestores, exceto no CISBAF, onde o tempo na gestão foi mais extenso Predominância de uma visão positiva, destacando a importância de uma APS robusta e abrangente, com valorização do modelo da ESF, visto como o arranjo mais efetivo Variações nas concepções segundo o grau de familiaridade com as diretrizes governamentais e os conteúdos abordados nos atos normativos federais sobre a APS Visão municipal positiva do Previne Brasil 2019, com foco no controle do desempenho dos serviços, mas com receios sobre a capacidade municipal de responder às novas demandas Preocupações com a perda de recursos financeiros federais e esforços da SES-RJ e COSEMS RJ para auxiliar os municípios Criticismo de alguns municípios à oposição SES/COSEMS ao Previne Brasil 2019, sugerindo uma abordagem de resolução dos problemas críticos sem buscar o encerramento político do programa	Maioria dos gestores municipais com formação na área da saúde, com trajetórias diversificadas e experiências em gestão prévia, em alguns casos relacionados a outros setores Representantes estaduais e regionais com qualificações avançadas, com ampla experiência em diferentes cargos de gestão municipal e/ou estadual O período na função gestora foi curto para a maioria dos gestores municipais, com exceção de um município, onde o gestor atuava na mesma função há pelo menos 20 anos Poucas variações nas concepções de APS entre os gestores municipais e as representações estaduais e regionais, com destaque para a importância desse nível de atenção Modelo da ESF entendido como fundamental para organizar a APS nos territórios, com exceção de três municípios Desconhecimento da maioria dos municípios sobre as alterações promovidas pela PNAB 2017 Predominância de um posicionamento favorável ao Previne Brasil 2019 entre os gestores municipais e representantes estaduais e regionais, mesmo reconhecendo os riscos de redução de repasse de recursos Pagamento por desempenho defendido por condicionar o repasse ao cumprimento de metas, obrigando os municípios a qualificarem os registros e reconhecimento da importância da capitação ponderada para a indução do cadastramento da população
Decisões relacionadas à organização do processo de trabalho na APS	Importância dos ACS reconhecida pelos municípios, mas polêmicas e opiniões divididas sobre aumento do piso salarial e regras de seleção baseadas em escolaridade Desenvolvimento de iniciativas municipais para preservar equipes de NASF após perda de incentivo federal, e compensação por meio de incentivo específico do Programa Estadual de Financiamento da Atenção Primária à Saúde pela SES-RJ Redução do número de profissionais nas equipes e deslocamento de trabalhadores para outros serviços de saúde, gerando conflitos com a lógica do núcleo Adoção de estratégias diversas para ampliação do cadastramento da população no e-SUS, envolvendo ACS e equipes Necessidade de qualificação dos registros de produção e preocupação com a qualidade da informação para cálculo de indicadores e captação de recursos federais Iniciativas variadas de capacitação dos profissionais de APS para qualificar registros de produção Implementação de apoiadores para solucionar problemas na imputação de dados de produção Uso de digitadores centralmente alocados nas secretarias municipais e sistemas eletrônicos contratados para suprir lacunas tecnológicas Fortalecimento dos processos de monitoramento e avaliação do desempenho dos profissionais de APS, com apoio técnico da SES-RJ e COSEMS RJ	Descontinuidade das equipes NASF em dois municípios, e manutenção de equipes em outro por meio de recursos próprios e contratação pelo consórcio Implantação do prontuário eletrônico, treinamento das equipes para o uso de sistemas informatizados e organização de novos fluxos de atendimento dos usuários Adoção de estratégias diversas para cadastramento da população, envolvendo ACS e equipes Falta de suporte técnico das Regionais de Saúde e do Ministério da Saúde como barreira Benefícios relatados nos municípios que adquiriram sistemas privados Implantação de instrumentos para monitoramento dos indicadores do Previne Brasil 2019 Responsabilidade pelo monitoramento: secretário de saúde, coordenação da APS ou enfermeira da EqSF Problemas identificados: falta de apoio técnico, dificuldade no uso do prontuário eletrônico e restrições no acesso à internet
Cobertura e composição da APS	Proporção média de população cadastrada na região menor, mas com variação percentual superior no período Ausência de municípios com 100% da população cadastrada no período Aumento de 13,1% no número de ACS na região Redução da média de ACS por EqSF e aumento da média de EqSF por UBS na região Número médio de EqAP no período menor do que o número médio de EqSF, mas com variação percentual expressiva nos municípios de ocorrência e na região	Proporção média de população cadastrada na macrorregião maior, mas com variação percentual inferior no período Presença de municípios com 100% da população cadastrada no SISAB no período Queda de 0,8% no número médio de ACS na macrorregião Redução mais acentuada (maior que 50%) da média de ACS por EqSF e aumento mais acentuado (maior que 100%) da média de EqSF por UBS na macrorregião Número médio de EqAP no período menor do que o número médio de EqSF, mas com variação percentual expressiva nos municípios de ocorrência e sem variação na macrorregião

ACS: agentes comunitários de saúde; APS: atenção primária à saúde; CISBAF: Consórcio Intermunicipal de Saúde da Baixada Fluminense; COSEMS: Conselho de Secretários Municipais de Saúde; EqAP: equipes de atenção básica; EqSB: equipes de saúde bucal; EqSF: equipe de saúde da família; NASF: Núcleos de Apoio à Saúde da Família; PNAB: Política Nacional de Atenção Básica; SES-RJ: Secretaria SISAB: Sistema de Informação em Saúde para a Atenção Básica; UBS: unidade básica de saúde.

Fonte: elaboração própria.

Resumidamente, a comparação da cobertura e composição da APS identifica três semelhanças e três diferenças entre as regiões. Quanto às semelhanças: (1) redução da média de ACS por EqSF e aumento de EqSF por UBS; (2) prevalência de EqSF sobre EqAP, que teve crescimento expressivo; e (3) padrões semelhantes na expansão de EqSB

Quanto às diferenças: (1) a Metropolitana I do Rio de Janeiro apresentou menor proporção de população cadastrada com maior variação percentual, e maior cobertura com menor variação na Macrorregional Norte do Paraná; (2) o número de ACS aumentou 13,1% na Metropolitana I do Rio de Janeiro, ao passo que, na Macrorregional Norte do Paraná, registrou-se uma redução de 0,8%; e (3) a Metropolitana I do Rio de Janeiro expandiu equipes NASF, enquanto a Macrorregional Norte do Paraná registrou redução em alguns municípios.

## Discussão

A análise evidenciou semelhanças e diferenças nas regiões. Observou-se mais semelhanças no perfil e nas concepções dos gestores municipais (eixo 1); mais diferenças nas decisões sobre o processo de trabalho (eixo 2); e equilíbrio na cobertura e composição da APS (eixo 3), influenciados pelo perfil dos municípios e contexto regional.

Na formação e trajetória dos gestores, a maioria possuía ensino superior em saúde e formação de pós-graduação em Saúde Coletiva, o que tende a favorecer a organização da APS ^21^. Não tinham formação em saúde 40% dos secretários municipais na Macrorregião Norte do Paraná, ao contrário da região Metropolitana I do Rio de Janeiro, favorecida pela maior concentração de universidades e cursos de pós-graduação de Saúde Coletiva.

A experiência prévia em gestão pública e o tempo no cargo foram semelhantes entre os entrevistados das regiões. No entanto, os secretários e coordenadores da Macrorregional Norte do Paraná mostraram menos anos de experiência na gestão em comparação aos do Rio de Janeiro, possivelmente devido à escassez de força de trabalho em saúde ^12^ e à alta rotatividade de gestores em municípios pequenos ^22^.

Há convergência nas concepções de APS e ESF entre os entrevistados. Desde a segunda metade dos anos 1990, a ESF consolidou-se como referência para a organização da APS no SUS ^2^, promovendo a disseminação de uma APS abrangente, impulsionada por ações de formação e capacitação. As mudanças recentes, marcadas pela menor indução federal do modelo ESF ^16^ ^,^ ^17^ ^,^ ^18^, encontram gestores locais que, apesar do pouco tempo no cargo, defendem a ESF. Contudo, o curto tempo de atuação gestora explica o silêncio quanto às mudanças da PNAB 2017. Ressalta-se que a aprovação dessa política contou com apoio de gestores municipais, sem grandes tensões quanto à agenda do Ministério da Saúde ^23^, pois criava condições para atender à demanda por repasses específicos para outras modalidades além das EqSF.

As regiões apresentaram aumento no número de EqSF, evidenciando a consolidação da ESF. Por sua vez, mesmo que a substituição de EqSF por EqAP tenha sido limitada por normativa do Ministério da Saúde ^24^, observou-se crescimento acentuado das EqAP, sugerindo-se mudança de modelo assistencial ^16^.

A percepção favorável sobre o Previne Brasil 2019 nas duas regiões reflete alinhamento político e expectativa de maior controle da gestão municipal sobre a produção de indicadores e cadastramento de usuários. Na região Metropolitana I do Rio de Janeiro, essa perspectiva foi interpelada pelos riscos de descontinuidade na alocação de recursos para a APS, apontados no movimento do COSEMS RJ em articulação com a SES-RJ, embora alguns municípios tenham questionado as intenções dessa mobilização. Na Macrorregional Norte do Paraná, diferentemente, mesmo com preocupações sobre a perda de recursos, prevaleceu o endosso dos representantes regionais/estaduais à visão favorável ao Previne Brasil 2019 em atender tais expectativas.

A maior convergência de concepções entre os entrevistados pode estar associada a diversos fatores: valorização nacional dos atributos da APS e ESF, expansão da perspectiva gerencialista na saúde pública, sobretudo pelo crescimento da terceirização da gestão de serviços do SUS para organizações sociais e empresas ^25^, os desafios enfrentados pela ESF em municípios de grande porte populacional, e a influência da indução federal sobre municípios de menor porte, por meio de incentivos financeiros e estratégias de formação para implementação de políticas estratégicas.

A principal divergência está relacionada à formação mais sólida dos gestores do Rio de Janeiro e à postura crítica ao Previne Brasil 2019 do COSEMS RJ e da SES-RJ. Ainda assim, a concepção de APS ampliada, mencionada pelos entrevistados das regiões, mostrou-se insuficiente na contraposição às diretrizes nacionais estabelecidas.

A redução no total de ACS em metade dos municípios analisados, entre 2017 e 2022, acompanhada da redução de ACS por equipe, refletem a inflexão na gestão federal, com menor ênfase no trabalho de base territorial e comunitária da ESF ^26^. Segundo Freire et al. ^27^, as alterações introduzidas pela PNAB 2017 evidenciam um processo de desvalorização do trabalho dos ACS em municípios com melhores condições socioeconômicas, acompanhado da ênfase no cuidado médico individual. Esses aspectos, somados à regulamentação do piso salarial da categoria em um contexto de restrição dos repasses federais, ajudam a explicar a redução observada. No entanto, esse cenário contrasta com evidências de correlação positiva entre os investimentos em programas de ACS e a ampliação do acesso, a redução das desigualdades e a melhoria da eficiência dos sistemas de saúde ^28^.

Ademais, o aumento de equipes por UBS alinha-se à mesma direção, sobretudo na Macrorregional Norte do Paraná, onde essa concentração pode contribuir mais acentuadamente para menor capilaridade da ESF, ampliando distâncias dos usuários e prejudicando o acesso, diferentemente da Metropolitana I, que apresenta alta densidade demográfica. A ausência de crescimento de UBS na mesma proporção das EqSF também pode ampliar desigualdades no acesso. A expansão de unidades ajuda a reduzir vazios assistenciais e facilita o acesso em áreas distantes. Em contrapartida, concentrar equipes em unidades existentes pode sobrecarregar o trabalho e potencialmente reduzir a qualidade do atendimento.

No que se refere às mudanças nos processos organizacionais da APS, observou-se que, em ambos os casos, os entrevistados atribuíram valor a iniciativas voltadas para o cadastro populacional e à definição de metas de desempenho, diretamente relacionadas à gestão dos trabalhadores e à organização dos processos de trabalho. Houve aumento da população cadastrada no SISAB nas duas regiões, percentualmente mais expressivo na Metropolitana I do Rio de Janeiro. Esses resultados estão em consonância com Sellera et al. ^29^, que identificaram aumento de 76,1% dos cadastros dos municípios entre 2019 e 2021, independentemente do porte populacional. Ressalta-se, contudo, que esse crescimento pode decorrer da migração de registros de outras plataformas para o e-SUS, de ajustes em cadastros preexistentes e da introdução de incentivos financeiros pelo Previne Brasil (2019), não refletindo, necessariamente, ampliação do acesso ou melhoria na qualidade da assistência. A variação percentual mais elevada no Estado do Rio de Janeiro está associada a um número inicial menor de cadastros, permitindo maior potencial de crescimento. Ademais, características regionais, como porte populacional dos municípios, implicam maior esforço de cadastramento nas grandes cidades ^29^.

Destaca-se a percepção de que a melhoria da gestão na APS depende do controle do processo de trabalho dos profissionais, vistos por alguns como instrumentos para atingir metas preestabelecidas, visando recompensas financeiras aos municípios. Entretanto, a ênfase no gerencialismo, focada em dados mensuráveis em detrimento dos cuidados integrais à população, não resulta necessariamente em avanços nos indicadores de resultados em saúde ^29^. Mendes & Carnut ^30^ (p. 205) ressaltam esse movimento como um processo de “*super intensificação do trabalho individual, por meio de estratégias de controle da produção, utilizando técnicas administrativas que incutem no trabalhador um autocontrole de si*”, desconsiderando, assim, as características coletivas inerentes ao trabalho em saúde.

O financiamento das equipes multiprofissionais é considerado uma estratégia indutora significativa para fortalecer o trabalho interprofissional ^31^. No Estado do Rio de Janeiro, a presença de um programa da SES de cofinanciamento à APS permitiu mitigar o impacto da perda de incentivo federal ao NASF, tendo a Metropolitana I ampliado em 23,8% o número de equipes. Em contraste, no Paraná, a responsabilidade pela continuidade do NASF recaiu sobre os municípios, observando-se a redução de 1,4% no número de equipes na Macrorregional Norte do Paraná.

A implementação das mudanças nas duas regiões evidenciou adesão ao cadastramento pela capitação ponderada e produção de informações para os indicadores do Previne Brasil 2019. Contudo, as abordagens adotadas variaram, dados os contextos locorregionais: no Rio de Janeiro, o papel dos ACS foi reconhecido, mas estavam presentes tensões com a gestão, o que interferiu no alcance das metas para incentivo financeiro; já no Paraná, os municípios ampliaram os cadastros com apoio tecnológico aos agentes. Estudo anterior já apontava que o trabalho dos ACS no Rio de Janeiro era marcado por “*múltiplas tensões, questionamentos e contradições*” em resposta ao modelo gerencialista ^32^ (p. 14). Apesar das diferenças, ambos os estados aumentaram cadastros.

As estratégias e instrumentos de monitoramento e avaliação da APS diferiram entre os casos. Quanto à qualificação dos registros, no Rio de Janeiro, focou-se na capacitação de profissionais da APS, apoio técnico da SES-RJ, ajustes centralizados nas secretarias municipais e uso majoritário do e-SUS. No Paraná, sistemas privados predominaram pela ausência de apoio técnico estadual, gerando custos adicionais e expondo ainda mais o SUS a interesses mercantis de venda de software e assessorias para gerenciamento de dados. Dessa forma, as decisões vinculadas ao Previne Brasil 2019 resultaram em rearranjos gerenciais e introdução de elementos de gestão empresarial com perspectiva produtivista, semelhantes aos adotados em outros municípios ^33^.

As regiões promoveram mudanças organizacionais na APS, em geral para atendimento às condicionalidades dos repasses financeiros. Contudo, diferiram nas estratégias para a manutenção de programas e ações estratégicas, como o NASF, e nas práticas de monitoramento e avaliação. Essas diferenças associam-se a fatores contextuais: no Rio de Janeiro, uma região metropolitana com municípios de grande porte, maior capacidade institucional e suporte técnico e financeiro estadual; no Paraná, uma macrorregião majoritariamente composta por municípios menores, mais dependentes das transferências federais para a APS e sem cofinanciamento estadual.

Entre as limitações do estudo, destacam-se os desafios de se analisar uma política em curso no momento da pesquisa, com mudanças na implementação e adiamento de regras. Isso inclui, especialmente a partir de 2020, as repercussões da pandemia de COVID-19, que impactou o financiamento e a organização dos serviços. Os dados do CNES estão sujeitos ao cadastramento inadequado, incompleto ou desatualizado, podendo, eventualmente, haver cadastro de equipes que não estão com a composição profissional completa e unidades fora de funcionamento.

## Considerações finais

Este estudo identificou fatores condicionantes, internos e externos aos contextos regionais e locais, que moldam a implementação e as repercussões das mudanças nas diretrizes federais da APS no Brasil.

Diferenças regionais ligadas ao porte populacional dos municípios e sua dependência aos recursos federais afetam capacidades e decisões organizacionais. As relações intergovernamentais, envolvendo aspectos administrativos, políticos e técnicos, e a atuação dos governos estaduais também interferem na adesão dos governos municipais às diretrizes nacionais. As repercussões variam com a cobertura prévia da ESF, com maior resistência em regiões mais cobertas. Esses elementos mostram que a implementação das diretrizes da APS opera em um contexto multifatorial, de relações complexas e interdependentes, definindo limites e possibilidades de transformação do sistema de saúde local.

A análise das regiões estudadas, com características contrastantes e especificidades no processo local de implementação, revela um ponto comum: o apoio e a adesão dos gestores municipais às mudanças nas diretrizes nacionais, sob uma perspectiva gerencialista. Esse movimento, impulsionado por políticas nacionais voltadas para a APS no período analisado, impõe desafios à garantia da qualidade e da integralidade do cuidado em saúde no SUS.
